# Electronic Journals: A New Dimension To Research

**Published:** 2012-01-01

**Authors:** Maham Zaman

**Affiliations:** Department of Pediatric surgery, The Children's Hospital and the Institute of Child Health Lahore, Pakistan

**Dear Sir,**

Ever since the humans have evolved the evolution is not limited to physical aspect of life but also to the world surrounding it. As man started to cope up with the chal­lenges of life he learnt new ways and methods. These ways opened doors to a whole new dimension of life- the research. Practice led to strange new discoveries and eventually the implementation of these moved us into better understanding. Technology, science, health and various other disciplines of life started to grow. With the advent of the 20th century mankind stepped into a whole new space, the “cyber space”.

The latest discovery in the field of writing has been the “electronic publication”. This mode of publication is now being used in all fields of research. Easily accessible, these electronic journals (e-journals) are available worldwide through the internet. Studies have been con­ducted to verify whether this entity is beneficial or harmful to the field of research. One such study con­ducted by the “Max Planck society” reveals that elec­tronic journals are far better than print journals. They suggest that round the clock downloading and easy access are one of the key reasons of their superiority over the print journals [1].

E-journals have a number of advantages. Among the long list, open access (many), free of cost abstracts, round the clock availability, and cost effectiveness are some points that are attractive to researchers. Another positive aspect of electronic publications is no space constrains for the content to be published and even videos can be incorporated in the manuscripts for better presentation of the research.

Many of these e-journals are usually indexed with many abstracting and indexing databases like Google Scholar, Pubmed, and Index Copernicus; therefore can easily be searched through the search engine sites without wast­ing much time. In conclusion, it is best to state that electronic journals are a new dimension to research. They not only ease the publications process but also improve its worldwide visibility.

## Footnotes

**Source of Support:** Nil

**Conflict of Interest:** None declared

